# Angiogenin in age-related macular degeneration

**Published:** 2011-02-23

**Authors:** Jessica M. Skeie, Shemin Zeng, Elizabeth A. Faidley, Robert F. Mullins

**Affiliations:** Department of Ophthalmology and Visual Sciences, University of Iowa Carver College of Medicine, Iowa City, IA

## Abstract

**Purpose:**

Age-related macular degeneration (AMD) is a common blinding disease in the elderly population. AMD is frequently complicated by choroidal neovascularization, causing irreversible losses in visual acuity. Proteins that induce pathologic angiogenesis in other systems include angiogenin, a small protein involved in angiogenesis in tumor metastases. Our goal was to determine if angiogenin participates in angiogenesis during choroidal neovascular membrane formation in AMD.

**Methods:**

The expression of angiogenin in the human retina and retinal pigment epithelium (RPE)-choroid was determined using reverse-transcription (RT)-PCR and immunoblotting. Localization of angiogenin in human control eyes and in eyes with choroidal neovascularization was determined using immunohistochemistry. Potential angiogenin-mediated effects on endothelial cell migration, as well as angiogenin internalization by Rf/6a cells, were determined.

**Results:**

Angiogenin was synthesized by the human choroid and retina and localized to normal and pathologic vasculature. Angiogenin did not change the migratory behavior of Rf/6a chorioretinal endothelial cells; however, these cells did internalize exogenous angiogenin in culture.

**Conclusions:**

Chorioretinal endothelial cells bind and internalize angiogenin, a protein localized to the choroid in normal eyes, as well as in some drusen and in neovascular membranes in AMD eyes. Angiogenin has been shown to participate in angiogenesis in other tissues. Although angiogenin does not increase the migratory behavior of these cells, it may play a role in other aspects of endothelial cell activation in neovascular AMD.

## Introduction

Age-related macular degeneration (AMD) is currently the leading cause of irreversible vision loss in the western world [1]. This disease is characterized by commonly described landmarks including drusen and retinal pigment epithelial atrophy [2–5]. Whereas many patients do not advance beyond early atrophic AMD, AMD can be complicated by end-stage, severe attenuation of the retinal pigment epithelium (RPE), termed geographic atrophy, or by abnormal blood vessel growth from the choroid into the RPE and/or the subretinal space [5–11]. This complication of AMD, referred to as choroidal neovascularization (CNV), severely decreases visual acuity, and is characterized by abnormal angiogenesis.

Numerous molecular pathways affect different facets of angiogenesis, and may therefore be involved in promoting neovascularization in human patients with AMD [12]. Vascular endothelial growth factor (VEGF)-signaling is the best-characterized of these, and the recent development of anti-VEGF therapy to treat patients with advanced AMD has been remarkably successful [13–15]. Other mitogenic polypeptides potentially involved in neovascular AMD may include fibroblast growth factor-1 (FGF1), FGF2, transformin growth factor (TGF)-β, or angiopoietins 1 and 2 [16–19]. An improved understanding of other molecules that may regulate angiogenesis in the human choroid is important for the development of additional therapies, especially if anti-VEGF therapies are found to have other, unappreciated, ocular, or systemic effects in some patients [20,21]. One protein that may be involved in this process is angiogenin, a small polypeptide that shares one third of its amino acid sequence with the enzyme, pancreatic RNase (RNase) A 1 [22]. Although the enzymatic activity of angiogenin is limited, it has been shown to promote angiogenesis in other systems [23–25]. We sought in this study to determine the localization and physiologic effects of angiogenin in the choroid, and to relate these findings to the development of neovascular membranes in AMD.

## Methods

### Angiogenin reverse-transcription PCR in human choroid and retina

Human donor eyes (n=3) were obtained from the Iowa Lions Eye Bank (Iowa City, IA) within 5 h of death (eye ages were 80, 84, and 87 years). All donor families fully consented, and all experiments were performed in accordance with the Declaration of Helsinki. The tissues used for reverse-transcription (RT)-PCR did not exhibit any sign of AMD. RNA from temporal, extramacular punches of RPE-choroid and retina was isolated separately, using the RNeasy mini-kit according to manufacturer’s instructions (Qiagen, Valencia, CA). Reverse transcription was performed using the High-Capacity cDNA Reverse Transcription kit (Applied Biosystems, Foster City, CA). For RT–PCR, cDNA was pooled from three RPE-choroid punches. PCR was performed (Biolase DNA polymerase kit; Bioline, Taunton, MA) using the following conditions for 35 cycles: denaturing at 94 °C for 45 s, annealing at 62 °C for 30 s, and elongation at 72 °C for 1 min. The angiogenin primers used were designed from the Ensembl #ENSG00000214274 sequence as follows: forward primer, 5′-CGT CCG TGT ACA CAC ACT CA-3′, and reverse primer, 5′-GCA CGA AGA CCA ACA ACA AA-3′ (Integrated DNA Technologies, Coralville, IA). PCR products were separated on a 2% agarose gel (FisherBiotech horizontal electrophoresis system; Fisher Scientific, Pittsburgh, PA) at 135 V for approximately 45 min. Gels were stained using ethidium bromide.

### Angiogenin detection with immunoblotting

Retinal and RPE/choroid tissue from the extramacular, temporal region of 3 human donor eyes was snap frozen in liquid N_2_ within 5 h post mortem. Tissue was homogenized in PBS with 1% Triton X-100 and complete protease inhibitor (Roche, Indianapolis, IN). Recombinant angiogenin (5 ng; R&D Systems, Minneapolis, MN) or 20 µg of choroid or retinal protein was mixed with an equal volume of 2× Laemmli buffer (BioRad Laboratories, Inc., Hercules, CA), boiled for 5 min, and then separated electrophoretically on a 4%–20% Tris-HCl gradient sodium dodecyl sulfate (SDS) polyacrylamide gel as described previously [26]. Next, proteins were blotted onto a polyvinylidene difluoride (PVDF) membrane (BioRad Laboratories, Inc., Hercules, CA). Following blocking in BSA (BSA), an antibody directed against angiogenin (R&D Systems) was employed at a concentration of 0.4 µg/ml, followed by washing of the membrane, incubation with 0.4 µg/ml peroxidase-conjugated species-specific secondary antibody (Santa Cruz Biotechnology, Santa Cruz, CA), and detection using the ECL Plus kit (Amersham Biosciences, Buckinghamshire, UK). As a loading control, anti-beta-actin antibody (Santa Cruz Biotechnology) was used on blots that were stripped with 10% SDS, 0.7% beta mercaptoethanol, 60 mM Tris Cl (pH 6.8) at 55 °C for 30 min.

### Angiogenin localization using immunohistochemistry

Sections of 22 human donor eyes ranging from 60 to 90 years of age, spanning from the macula to the ora serrata and containing both retina and RPE/choroid, were collected and fixed in 4% paraformaldehyde for 2 h within 8.5 h, post mortem. All eyes were obtained from the Iowa Lions Eye Bank with consent, as stated above. Eyes included two samples with CNV and three with early AMD. The tissues were infiltrated in increasing concentrations of sucrose (up to 20%) and then embedded in two parts 20% sucrose to one part optimal cutting temperature medium (Sakura, Torrance, CA) [27]. Frozen sections at a thickness of 7 µm were collected using a cryostat.

Sections were blocked in 1% horse serum for 15 min. The primary antibody used, at a concentration of 4 µg/ml, was an antibody to human angiogenin raised in rabbits (Santa Cruz Biotechnology). Immunolabeling was performed as described previously [28]. Briefly, sections were incubated with anti-angiogenin antibody for 1 h, rinsed, probed with biotinylated secondary antibody and avidin-horseradish peroxidase complex, and visualized using the Vector VIP development kit according to manufacturer's instructions (Vector Laboratories, Burlingame, CA)

### Assessment of angiogenin-mediated choroidal endothelial cell migration

To determine whether angiogenin induces migration of endothelial cells from the posterior pole, Rf/6a cells (a primate chorioretinal endothelial cell line, American Type Culture Collection, Manassas, VA) were seeded into the upper chamber of 8 μm pore culture inserts (Millipore, Billerica, MA) within a 24-well plate. Recombinant human angiogenin protein was added to the lower chamber, diluted in serum-free medium at a concentration of 0.5 μg/ml, which is 2× the normal human serum concentration [29]. Elastin derived peptides were used at a concentration of 1.0 μg/ml as a positive control [30] and an equal volume of protein diluent (Hank's Buffered Salt Solution-HBSS) was used as a negative control. All experiments were performed in triplicate. Cells were allowed to migrate for 8 h. Cells that migrated to the lower surface of the insert were counted using scanning electron microscopy (SEM) as described previously [30]. Statistical analysis was performed using Student’s *t*-test.

### Angiogenin uptake by chorioretinal endothelial cells

The internalization of angiogenin by Rf/6a cells was determined. Cells were cultured in F12 medium supplemented with 1% fetal bovine serum (FBS), 20 ng/ml bFGF [31], and penicillin/streptomycin on glass coverslips for 24 h. The cultures were not allowed to reach confluency, to maximize the internalization of protein. Recombinant human angiogenin protein (1 µg/ml) or 0.1% BSA control protein was added to the medium of the cultures for 24 h. Cells were then fixed for 10 min in 4% paraformaldehyde (pH 7.4), and immunocytochemistry was performed using an antibody directed against angiogenin (Santa Cruz Biotechnology) as described above for tissue sections, except that Alexa-546 conjugated secondary antibodies (Invitrogen, Eugene, OR) were used to detect angiogenin. Phalloidin (Invitrogen, Eugene, OR) was added to the secondary antibody incubation to visualize filamentous actin. Cells were imaged using an Olympus BX41 microscope (Olympus, Tokyo, Japan) equipped with fluorescent filter sets and a SPOT-RT camera (Diagnostic Instruments; Burlingame, CA). Independent images for angiogenin, actin, and diamidino-2-phenylindole (DAPI) were collected separately and merged.

## Results

### Expression of angiogenin in human retina and choroid using PCR

**Figure 1:**
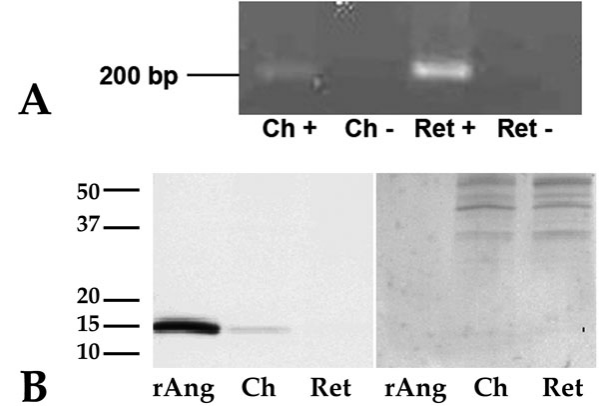
Angiogenin is expressed in the human retina and retinal pigment epithelium (RPE)-choroid. **A**: Reverse-transcription (RT)-PCR results. Angiogenin cDNA was amplified from both human retina (Ret) and choroid (Ch) tissues. Experiments were performed with (+) and without (−) reverse transcriptase enzyme, the latter serving as controls. **B**: western blot results show that the angiogenin protein is detectable only in the retinal pigment epithelium-choroid samples (left panel). Recombinant human angiogenin protein (rAng) was detected at the correct molecular weight (14 kDa) with the anti-angiogenin antibody as a positive control. The stripped blot, probed with anti-actin antibody (right panel) shows even loading between retinal and choroidal samples.

To determine if the gene for angiogenin was expressed in the human retina and choroid samples, non-quantitative PCR was performed. Reverse-transcribed mRNA transcripts for the angiogenin gene were amplified in both the retina and choroid samples (Figure 1A).

### Angiogenin protein expression in human retina and choroid using immunoblotting

Western blots of human RPE-choroid protein probed with anti-angiogenin antibody showed the expression of a single immunoreactive band at the expected molecular weight of 14 kDa (Figure 1B) and at the same position on the blot as recombinant protein. In contrast to its mRNA, angiogenin protein was not detected in the retina samples using immunoblotting.

### Localization of angiogenin in human retina and choroid

**Figure 2:**
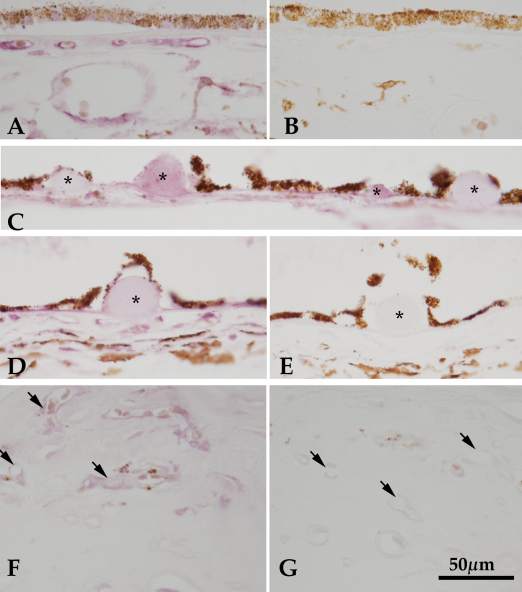
Angiogenin localization in human eyes. Labeling of angiogenin is shown in a normal macular choroid (**A**), in which angiogenin is expressed on the endothelial cells of the choriocapillaris and large vessels, as well as localized to intercapillary pillars. Modest anti-angiogenin labeling is detected in some extramacular drusen (**C** and **D,** asterisks). In some donors, approximately 50% of the hard drusen showed weak immunoreactivity (**C**). Minor labeling of a hyaline druse (**D**, asterisk) was observed in an 83-year-old female with neovascular age related macular degeneration (AMD), compared to an adjacent section in which the primary antibody was omitted (**E**). Endothelial cells in a neovascular membrane (**F**) from the macula of human donor eye with advanced AMD were also reactive with anti-angiogenin antibody. The presence of angiogenin in the neovascular membrane vessels indicates that it may play a role in the pathophysiology of these cells in AMD. Controls in which primary antibodies are omitted are shown for normal tissue (**B**), drusen (**E**), and neovascular tissue (**G**). Scale bar represents 50 μm.

To determine the cells responsible for endogenous expression of angiogenin in the human choroid samples, anti-angiogenin immunohistochemistry was performed. In labeled sections, the choriocapillary pillars were positive, as well as capillary endothelial cells and endothelial cells in larger choroidal vessels (Figure 2A). This pattern indicated that the angiogenin peptide was present in the human choroid in vivo. Sections that were not exposed to primary antibodies showed no labeling (Figure 2B). There was variable labeling of drusen in sections from patients with AMD. Many soft drusen (i.e., large drusen with sloping borders that were >63 µm) had very sparse, punctate labeling (not shown), while nodular drusen either had moderate labeling or no labeling (Figure 2C,D,E). Tissues from patients with neovascular AMD exhibited angiogenin labeling on the endothelial cells in the choroid, as well as on those within the neovascular membranes (Figure 2F,G).

### Effects of angiogenin on chorioretinal endothelial cell migration

**Figure 3:**
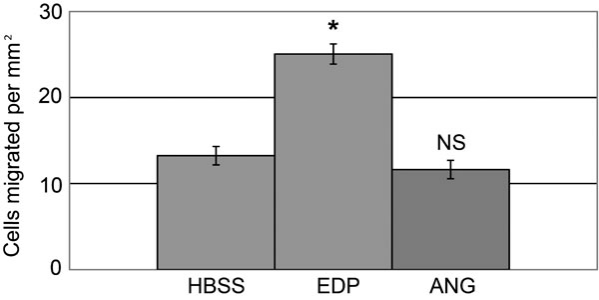
Angiogenin does not promote migration of chorioretinal endothelial cells. Angiogenin protein (ANG) did not promote endothelial cell migration, and migration was comparable to that observed with Hank's Buffered Salt Solution (HBSS) alone. The positive control, elastin derived peptides (EDP), resulted in a marked increase in migration as noted previously [30]. Asterisk (*) indicates a p value of <0.01. NS means not significant.

A Boyden’s chamber migration assay was used to measure the changes in migration of chorioretinal endothelial cells in the presence of elevated levels of angiogenin protein. The presence of angiogenin at the tested concentration did not affect the migratory response of chorioretinal endothelial cells in this assay (Figure 3). The *t*-test p value was 0.3. Exposure of EC to the positive control, elastin fragments, resulted in a significant increase in migration compared to the control, as previously described [30] (p<0.01).

### Nuclear translocation of angiogenin by chorioretinal endothelial cells

**Figure 4:**
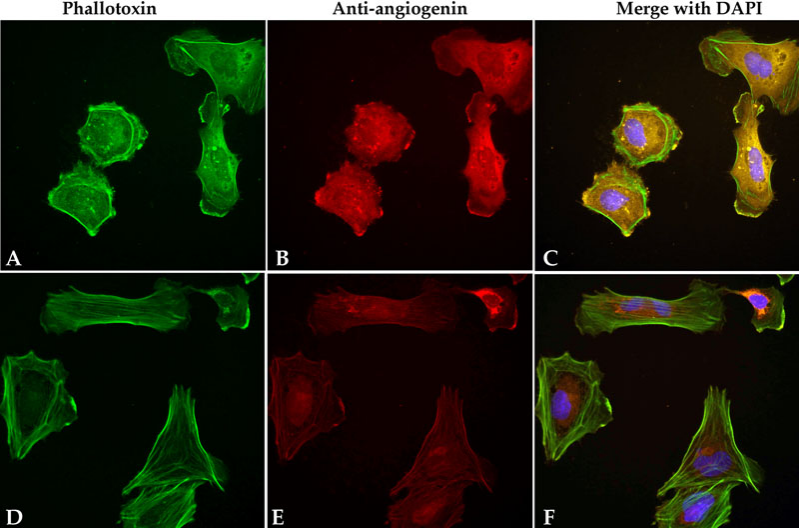
Endothelial cells internalize angiogenin in culture. Rf/6a chorioretinal endothelial cells incubated with recombinant human angiogenin (**A**-**C**) and with the control protein, BSA (BSA; **D**-**F**). Cells exposed to angiogenin exhibit intense angiogenin labeling, indicating that they actively import the protein. Cells that were not exposed to angiogenin demonstrated only faint labeling in the cytosol, attributed to endogenous angiogenin. Filamentous actin is labeled with phalloidin (**A**, **D**) and nuclei are labeled with diamidino-2-phenylindole (DAPI; blue).

Chorioretinal cells that were incubated with the recombinant human angiogenin protein showed significantly higher angiogenin labeling within the cytoplasm and the nucleus than within the control cells that had been pre-incubated with BSA only (Figure 4), indicating that these cells were capable of angiogenin uptake. Cells in both experimental groups demonstrated some labeling of angiogenin due to the presence of endogenous protein.

## Discussion

The activation of endothelial cells and the development of choroidal neovascular membranes in patients with AMD are complex events that are not well defined. Although there are very effective anti-VEGF treatments currently in use for the treatment of existing neovascular membranes, the precipitating causes underlying the formation and growth of choroidal neovascular membranes (CNVMs) is likely to be complex. Determining these other events will further our knowledge of the causal mechanisms occurring in AMD and will provide more complete therapeutic options.

We hypothesized that angiogenin may elicit neovascular behaviors in ocular endothelial cells. Although the role of angiogenin in neovascular eye diseases has not been established, its concentration in the vitreous of proliferative diabetic retinopathy patients has been shown to be elevated [32,33]. This finding indicates a possible role for angiogenin in the retinal neovascularization that occurs in diabetic retinopathy.

In our studies, angiogenin does not appear to increase migration—one measure of angiogenic behavior—in chorioretinal endothelial cells. The positive control for choroidal EC migration, elastin peptides, did promote migration as described previously [30], demonstrating that the EC were capable of responding to chemotactic stimuli. We did not assess other potential roles of angiogenin in functions associated with choroidal neovascularization, such as extracellular matrix turnover, tubulogenesis, or proliferation. It is possible that angiogenin participates in some, but not other, aspects of angiogenesis in the aging eye.

Chorioretinal endothelial cells were found to internalize angiogenin peptides from the microenvironment, suggesting that these cells express angiogenin receptor(s), which are yet to be conclusively identified [34,35]. In other systems, angiogenin exerts complex effects on cell behavior that include direct interaction with filamentous actin [36], B/Akt kinase activation, and rRNA synthesis [31]. Since Rf/6a cells are able to bind angiogenin and import it from their microenvironment, it is possible that this protein participates in the regulation of some aspects of inflammation and/or angiogenesis in the human choroid, central features of neovascular AMD.

Finally, we found that angiogenin is present within some drusen, which is intriguing since these deposits occasionally appear to be a substrate for angiogenesis (R.F.M., unpublished observations). Moreover, angiogenin was localized to both the normal vasculature and the pathologic endothelium in eyes with neovascular membranes, suggesting that angiogenin may be necessary in maintaining the vasculature and that angiogenin polypeptides are present during the pathologic development of AMD. A comprehensive understanding of angiogenin and its potential receptor(s) in normal and diseased human eyes, and its effects on endothelial cell physiology and biochemistry, may provide further insights into AMD disease progression.
